# Age-related vascular stiffening: causes and consequences

**DOI:** 10.3389/fgene.2015.00112

**Published:** 2015-03-30

**Authors:** Julie C. Kohn, Marsha C. Lampi, Cynthia A. Reinhart-King

**Affiliations:** Department of Biomedical Engineering, Cornell UniversityIthaca, NY, USA

**Keywords:** endothelial cell, atherosclerosis, pulse wave velocity, atomic force microscopy, collagen, glycation, mechanics, mechanical properties

## Abstract

Arterial stiffening occurs with age and is closely associated with the progression of cardiovascular disease. Stiffening is most often studied at the level of the whole vessel because increased stiffness of the large arteries can impose increased strain on the heart leading to heart failure. Interestingly, however, recent evidence suggests that the impact of increased vessel stiffening extends beyond the tissue scale and can also have deleterious microscale effects on cellular function. Altered extracellular matrix (ECM) architecture has been recognized as a key component of the pre-atherogenic state. Here, the underlying causes of age-related vessel stiffening are discussed, focusing on age-related crosslinking of the ECM proteins as well as through increased matrix deposition. Methods to measure vessel stiffening at both the macro- and microscale are described, spanning from the pulse wave velocity measurements performed clinically to microscale measurements performed largely in research laboratories. Additionally, recent work investigating how arterial stiffness and the changes in the ECM associated with stiffening contributed to endothelial dysfunction will be reviewed. We will highlight how changes in ECM protein composition contribute to atherosclerosis in the vessel wall. Lastly, we will discuss very recent work that demonstrates endothelial cells (ECs) are mechano-sensitive to arterial stiffening, where changes in stiffness can directly impact EC health. Overall, recent studies suggest that stiffening is an important clinical target not only because of potential deleterious effects on the heart but also because it promotes cellular level dysfunction in the vessel wall, contributing to a pathological atherosclerotic state.

## Introduction

Cardiovascular diseases are the leading cause of death worldwide ^1^, and age is considered a primary risk factor (Benetos et al., 2002). Arteries stiffen with both age and disease suggesting that age-related arterial stiffening may contribute to cardiovascular pathologies (Benetos et al., 2002). Macroscale analysis of composite arterial mechanics are widely used in the clinic, however, the importance of layer-specific microscale mechanics in altering cellular function to contribute to cardiovascular diseases is less well-established. In this review, we will discuss the structure and composition of the artery and each of its layers during aging as well as methods used to measure the mechanical properties of the artery at both the macro- and microscales. Recent advances in our knowledge of the effects of these mechanical changes on the cells within the vessel are discussed as well as limitations in the current clinical approaches to prevent and/or reverse vessel stiffening.

### Artery Structure

**FIGURE 1:**
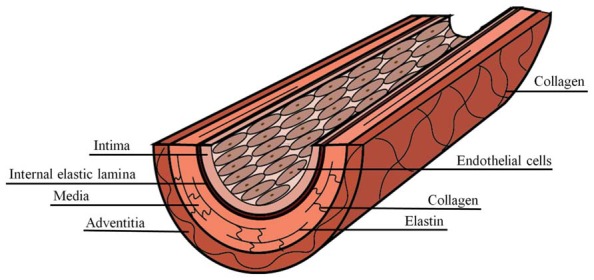
**Composite structure of the artery**.

Arteries are composite materials, containing multiple concentric layers, each with a distinct composition and function (**Figure 1**). The intima is the innermost artery layer and is a composite of two layers. The luminal layer, known as the basal lamina, is comprised of a thin basement membrane with a proteoglycan rich matrix and small amounts of collagen (Palotie et al., 1983; Kramer et al., 1984). Endothelial cells (ECs) attach to the intimal basement membrane and line the arterial lumen where they act, in part, as regulators of vascular homeostasis. The second intimal layer is musculoelastic and consists of elastin fibers, individual smooth muscle cells, and collagen (Stary et al., 1992). The intima is separated from the media, the inner layer of the artery, by fenestrated elastin fibers of the internal elastic lamina. Elastin within the internal elastic lamina is oriented longitudinally in the direction of luminal blood flow, while in the media it is oriented circumferentially (Farand et al., 2007). The media is composed of lamellar units that are composites of elastin fibers, circumferentially oriented vascular smooth muscle cell (VSMC) layers, collagen fibers, and a mucopolysaccharide viscoelastic gel, commonly referred to as a “ground substance” (Stary et al., 1992). The characteristic lamellae of the media comprise the majority of the arterial wall bulk and are responsible for its elastic properties, allowing for the artery to expand and contract with the blood pulse. The outermost artery layer is the adventitia, which is composed of circumferentially arranged, wavy collagen fibrils intermixed with elastin and is surrounded by loose connective tissue. Fibroblasts are dispersed within the adventitia, but are generally absent from the intima and media artery layers (Ross and Glomset, 1973).

### Arterial Mechanics

#### Composite Structure

When analyzed as a composite material, large arteries exhibit a non-linear stress-strain pattern, and therefore, are best described in terms of an elastic modulus evaluated at a given physiological stress along the stress-strain curve, termed the incremental elastic modulus (Bergel, 1961). The extracellular matrix (ECM) proteins collagen and elastin account for approximately half of the vessel dry weight, and play a crucial role in artery mechanics (O’Connell et al., 2008). Overall, Type I and Type III collagens account for 60% of the artery wall, and elastin 30% (Rizzo et al., 1989; Powell et al., 1992). At low degrees of stretch, the compliant elastin fibers dominate the mechanics, while at higher levels of deformation, helically oriented collagen fibers are recruited (Roach and Burton, 1957; Schriefl et al., 2012). Collagen fibers are 100–1,000 times stiffer than elastin which causes a sharp increase in the incremental elastic modulus at higher levels of circumferential stretch (Dobrin, 1978; Wagenseil et al., 2005; Lasheras, 2007; Wagenseil and Mecham, 2012). Under physiological strain loads, the incremental elastic modulus is a function of strain and the combined contributions of elastin and collagen (Wagenseil and Mecham, 2012).

The distinct composition of each artery layer lends itself to layer-specific mechanical properties that can vary from person to person, and also between large and small arteries (Baynes and Thorpe, 1999; Fonck et al., 2009). For example, measured under axial stretch and using non-axisymmetric deformations, the media is significantly less compliant than the adventitia (Yu et al., 1993; Pandit et al., 2005). The media and adventitia also have different load bearing proportions. Under circumferential tension, the media bears ∼60% of the load and the adventitia bears 40%. Longitudinal tension is primarily assumed by the adventitia which bears ∼75% of the load (Lu et al., 2004). Given that the mechanical properties of each layer vary, it is important to note that the cells within each layer are exposed to and sense the properties of the layer within which they reside. As such, measurements and analysis of each individual layer is essential when considering cellular level mechanobiology.

#### Intima

The mechanical properties of the healthy intima are not well-established largely because artery mechanics are traditionally studied on the macroscale, where properties of the media and adventitia dominate. Several studies have analyzed the aorta as a two-layer construct consisting of a combined intima-media layer surrounded by the adventitial layer (Holzapfel et al., 2000; Lu et al., 2003). Cells sense their local mechanical properties on the order of five microns, suggesting that overall or composite layer artery mechanics are not representative of the microenvironment where ECs reside (Sen et al., 2005; Buxboim et al., 2010). Interestingly, recent evidence has shown that ECs are mechanosensitive to matrix stiffness and that increased intimal stiffness promotes endothelial dysfunction; therefore the mechanical properties of the intima alone may be important factors in cardiovascular disease progression and warrant further investigation (Reinhart-King et al., 2005; Huynh et al., 2011). In prior work, our laboratory utilized atomic force microscopy (AFM) to measure the mechanical stiffness of the denuded aortic bovine intima, a composite of surface collagen and underlying elastin (Buxboim et al., 2010; Peloquin et al., 2011). The healthy human intima has been reported to have an elastic modulus of 34.4 kPa and is considered a compliant material, with an elastic modulus similar to adipose tissue and lower than muscle (Lundkvist et al., 1996; Engler et al., 2004; Patel et al., 2005).

#### Media

Medial mechanics are dominated by elastin within the lamellar units at physiological pressures (Greenwald et al., 1997). When the artery is subjected to a transmural pressure, the elastin fibers possessing high entropically driven recoil properties are initially stretched, followed by the stretching of stiffer collagen fibers (Samila and Carter, 1981). The number of concentric lamellae layers in the media remains constant with age but scales with arterial radius and vessel wall tensional strength (Wolinsky and Glagov, 1967). The mechanical contribution of VSMCs dispersed within the aortic lamellae is still somewhat unclear. Contractile activation of VSMCs has been shown to increase the medial elastic modulus (Dobrin and Rovick, 1969; Tremblay et al., 2010), but separate studies have shown that lamellar mechanical properties are unchanged after VSMC activation with noradrenaline (Berry et al., 1975). Therefore, the exact contribution of the VSMC to the mechanical properties of the medial layer is not yet well-defined.

#### Adventitia

The mechanics of the adventitia are attributed to the collagen organization. In the zero-load state, the fibers assume a crimped morphology (Rezakhaniha et al., 2012). As an axial load is placed on the vessel, the collagen fibers deform and straighten, exhibiting their high tensile strength. In the inner adventitia, collagen fibers are oriented, thin, and intermixed with elastin allowing for vessel distension and protection against rupture, whereas the outer adventitia is primarily composed of thick, non-oriented collagen fibers that support the vessel (Chen et al., 2011).

## Causes of Age-Related Arterial Stiffening

### Changes in Elastin

Elastic fibers have an extremely low turnover rate *in vivo,* and this longevity allows for the accumulation of age-related changes caused by fragmentation, calcification, and MMP-degradation (Schlatmann and Becker, 1977). As elastin fibers decay, they lose functionality and shift load bearing onto stiffer collagen fibrils, which directly contributes to significant increases in arterial stiffness. Fatigue failure from pulsatile wall stress can cause elastin fragmentation throughout the lifetime (O’Rourke, 1976; Avolio et al., 1998; Greenwald, 2007). Calcium in the arterial wall also increases with age facilitating the direct binding of calcium ions to elastin fibers causing calcification (Urry, 1971; Urry and Ohnishi, 1974; Otto et al., 1999). Animal models that induce elevated elasto-calcinosis show increased medial elastin fragmentation and arterial stiffness (Elliott and McGrath, 1994; Gaillard et al., 2005).

Enzymatic degradation of elastin is mediated by matrix metalloproteinases which have low basal activity in healthy arteries to balance the absence of new elastin synthesis. With age, increased activity of the elastases MT1-MMP and MMP-2 has been observed, and MMP-2 has been found near fragmented elastin fibers within the aorta (Wang et al., 2003; Yasmin et al., 2005; Bonnema et al., 2007). The dysregulation of MMPs is already known to play a role in the cardiovascular pathologies hypertension and aneurysm (Thompson and Baxter, 1999; Yasmin et al., 2005). MMP-2 is found near fragmented elastin fibers within the aorta. Notably, even though the absolute elastin content in the aorta remains relatively stable with age, the elastin concentration decreases and is accompanied by a substantial increase in collagen concentration (Myers and Lang, 1946; Kanabrocki et al., 1960; Toda et al., 1980; Fonck et al., 2009). Age is also associated with changes on the amino acid scale that can contribute to decreased arterial compliance caused by a loss of elastin functionality. The compounds desmosine and isodesmosine are formed from four lysine amino acids and are critical for crosslinking elastin fibers to give them their elastic properties (Davis and Anwar, 1970). The concentrations of desmosine and isodesmosine and their crosslinks decrease with age (John and Thomas, 1972; Watanabe et al., 1996).

### Changes in Collagen

In contrast to elastin, the collagen concentration in all three layers of the arterial wall increases with age, shifting the elastin:collagen balance that governs healthy arterial mechanics. Medial fibrosis occurs as a consequence of collagen fibers replacing VSMCs (Schlatmann and Becker, 1977). In general, in individuals over the age of 50, collagen redistributes within the media to bundle near lamellae units (Schlatmann and Becker, 1977; Greenberg, 1986). Recently it was shown that aged ECs have morphological changes resembling a VSMC phenotype and express smooth muscle alpha actin and collagen I, indicating they may also deposit collagen that contributes to intimal thickening (Fleenor et al., 2012). Within the adventitia, collagen I and III deposition by fibroblasts increases with age and is accompanied by vessel stiffening (Fleenor et al., 2010).

In concert with increased collagen concentrations, collagen crosslinking by non-enzymatic glycation increases arterial stiffness with age (Sims et al., 1996; Schleicher et al., 1997). Glycation is a reaction between reducing sugars and proteins, and directly stiffens tissues in addition to producing deleterious end products. Advanced glycation end products (AGEs) accumulate through a Maillard reaction. Amino groups on proteins react with aldehydes or ketones on the reducing sugars to form shift bases that rearrange to Amadori products and are further modified to produce AGEs (Bakris et al., 2004; Sell and Monnier, 2012). The mechanism of AGE crosslinking *in vivo* is hypothesized to predominately occur between lysine residues on collagen and the AGE *N-ε-carboxy-methyl-lysine* (CML) (Ikeda et al., 1996; Schleicher et al., 1997). In addition to collagen crosslinking, AGEs are harmful to vascular helth because they reduce nitric oxide availability, an important vasodilator used to maintain vascular tone that also has anti-inflammatory effectors on the endothelium (Bucala et al., 1991; De Caterina et al., 1995; Xu et al., 2003, 2005). Furthermore, AGEs interact with the receptor for advanced glycation end products (RAGE) to have downstream effects that include the production of reactive oxygen species, NF-κB inflammatory signaling, and endothelial hyperpermeability (Mullarkey et al., 1990; Wautier et al., 1996, 2001; Bierhaus et al., 1997; Park et al., 1998).

## Techniques to Measure Vascular Stiffening

Given that age is a significant cardiovascular risk factor and that arterial stiffness is known to increase with age (Benetos et al., 2002), both macro- and microscale testing techniques have been developed to evaluate arterial mechanics. Arterial properties are measured on the macroscale to determine bulk mechanics, and on the microscale to determine layer-specific mechanics on the cellular level. Historically, greater emphasis has been placed on macroscale testing because it can be used clinically and is associated with cardiovascular events including aneurism, atherosclerosis, and hypertension (van Popele et al., 2001; Kaess et al., 2012; van Sloten et al., 2014). The emergence of ECM mechanics as an important cue affecting cell function has contributed to the development of new techniques to analyze microscale arterial properties.

### Macroscale Mechanical Tests

**FIGURE 2:**
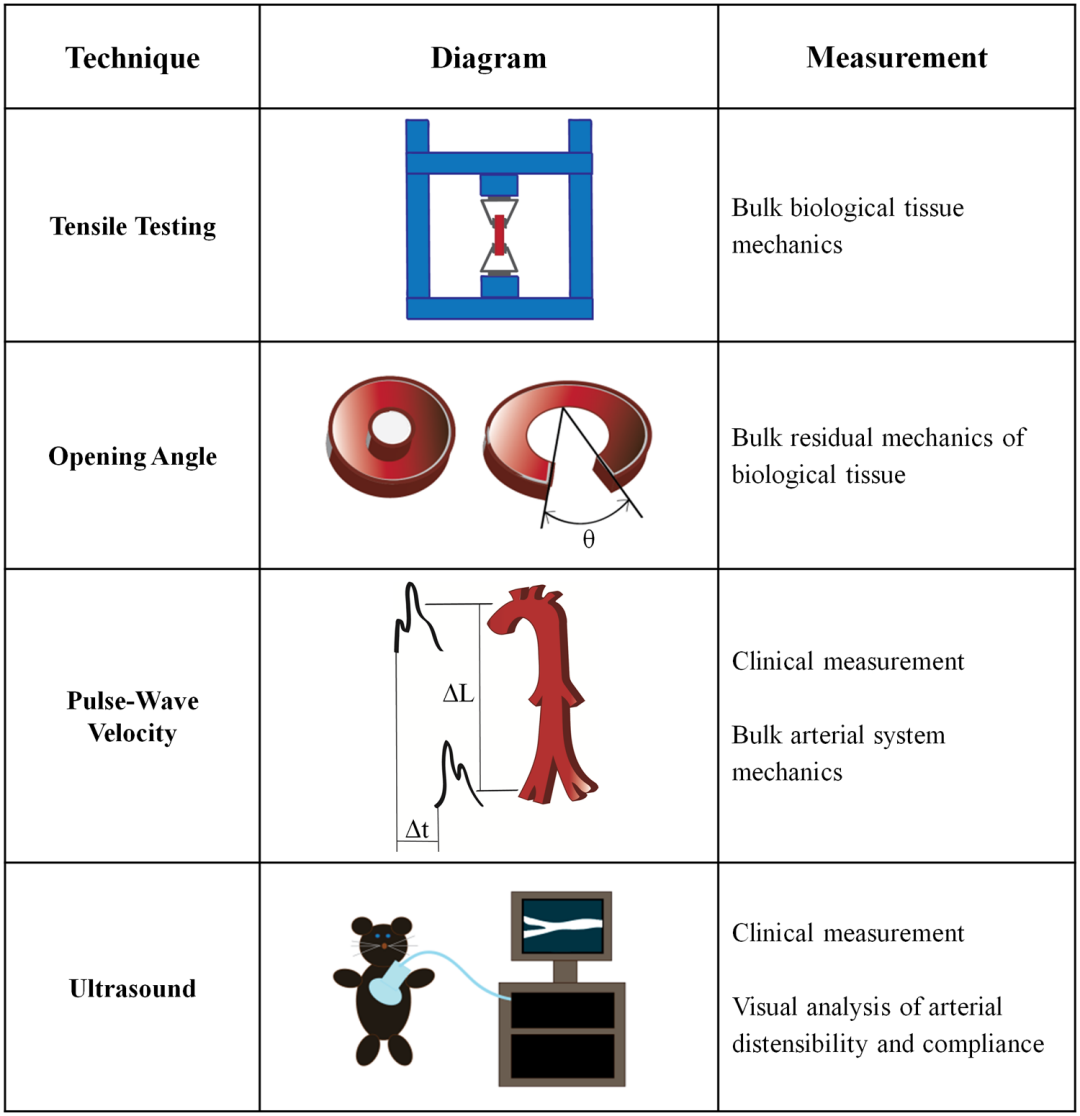
**Macroscale techniques to determine artery mechanics**.

Bulk mechanical testing on arteries is well-established and has been performed for over half a century (Hallock, 1934; Newman and Greenwald, 1978). Macroscale arterial mechanics are used clinically to predict the likelihood of cardiovascular disease risk (Blacher et al., 1999), and are also essential to the development of tissue engineered vascular grafts (Ravi and Chaikof, 2010). Several macroscale techniques for measuring the mechanical properties of intact arteries have been developed (**Figure 2**).

#### Tensile Testing

Tensile measurements are made by applying tension to a material until it reaches failure, producing a stress-strain curve. Artery samples are removed from the host, cut circumferentially or longitudinally, stored in temperature-controlled environments and tested within 48 h of surgery (Khanafer et al., 2013). As opposite ends of the sample are pulled vertically, the material enters the plastic regime and begins to exhibit necking, before ultimately reaching the failure point. The ultimate tensile strength, the elastic modulus (Young’s Modulus), yield strength and the maximum elongation of the bulk material can be obtained from a tensile test stress-strain curve. For materials with non-linear stress-strain patterns, the incremental elastic modulus value can be calculated at specific locations along the stress-strain curve. Many studies treat the artery as an isotropic material and measure its properties uniaxially, although it should be noted that anisotropic materials are most accurately tested biaxially (Sacks, 2000; Vande Geest et al., 2005).

The most common method to determine the elastic modulus of an artery is based on the quotient of stress and strain derived from tensile testing. Despite the widespread use of tensile testing, there are more than four definitions of stress and strain in the literature, which leads to differences in the values obtained through mechanical testing results (Khanafer et al., 2013). The most common values used are engineering stress, which is defined as the force applied to the original cross-sectional area, and engineering strain, which is defined as the change in length over the original length (Khanafer et al., 2013). The Cauchy stress, or force per deformed area, is often used in tensile analysis of arteries (Khanafer et al., 2011; Walsh et al., 2014). The stretch ratio, or the final length over the initial length, is another commonly considered property in the mechanical characterization of arteries when the artery demonstrates a large deformation under tension (Khanafer et al., 2013). Given the various definitions of stress and strain, both the measurement technique and the associated analysis should be considered to accurately compare the reported mechanical properties.

Tensile testing has been performed on human arteries to characterize their mechanical changes in a number of conditions, including aging (Vande Geest et al., 2005; Haskett et al., 2010; Shafigh et al., 2013), as well as plaque (Walsh et al., 2014) and aneurism development (Khanafer et al., 2011). Most often, aortas, coronary arteries, and carotid arteries are used to study the differential mechanics of diseased states after autopsy because they are the dominant sites of disease development, and as the largest vessels, they are also the easiest to obtain (Glagov et al., 1988; Atienza, 2010; Karimi et al., 2013; Walsh et al., 2014). Healthy human coronary arteries have an elastic modulus of 1.5 MPa, which increases to 3.8 MPa in atherosclerotic vessels (Khanafer et al., 2011). These values are comparable to moduli for patients with aneurisms, where the circumferential elastic modulus is 3 MPa and the longitudinal elastic moduli is 2.3–3.7 MPa, when measured under physiological pressures (Khanafer et al., 2011). Interestingly, biaxial tensile testing has also shown that individuals over the age of 30 have stiffer abdominal aortas compared to younger patients (Vande Geest et al., 2005). These data suggest that stiffening may precede significant disease progression.

Tensile testing is a common technique for measuring human artery mechanics because it allows for straight-forward experimentation and simple calculations. However, the artery in particular is a complex viscoelastic composite material, and therefore, proper mechanical testing should incorporate these attributes (García et al., 2012; Walsh et al., 2014). Recent experiments have used biaxial testing devices to study blood vessels (Vande Geest et al., 2005; Zemánek et al., 2009; Haskett et al., 2010), which are more representative of *in vivo* mechanical loading. As examples, biaxial testing has been used to microscopically observe the failure point of artery rupture (Sugital and Matsumoto, 2013) and to show that the arterial wall is stiffer in the circumferential direction compared to the axial direction (Shafigh et al., 2013).

#### Opening Angle Measurements

The opening angle (OA) is a measure of the residual stress of an unloaded tissue and has been used as an indicator of artery composition and viscoelasticity (Fonck et al., 2007; Zhang et al., 2008; Wan and Gleason, 2013). The OA measurement was originally developed to understand the stress-free state of tissues (Chuong and Fung, 1986) but can also be used to elucidate the viscoelastic properties of an artery after tissue loading (Rehal et al., 2006). The OA of a blood vessel is the angle formed from the tip of the open sector to the midpoint of the interior wall after the intact vessel is cut in the radial direction (Zhang et al., 2008). OA measurements are commonly used to study diseased arteries, especially with respect to a hypertensive state, because they are a direct indicator of residual vessel stress. To understand the elastic properties of the vessel, it is important to characterize the stress-free reference state (Fung et al., 1979; Chuong and Fung, 1986).

As examples, OA measurements have been used to measure the anisotropic response of arteries in hypertensive, diabetic and hypoxic conditions (Fung, 1990; Fung and Liu, 1991). Hypertension causes increased OA and conversely, arteries treated with a vasoconstrictor display a decreased OA (Han et al., 2006). OA is also correlated with systolic blood pressure (Matsumoto and Hayashi, 1996; Han et al., 2006) and with smaller-scale physiological changes. For example, increased OA correlates with an increase in VSMC proliferation and decreased OA values correlate with increased VSMC contraction (Han et al., 2006).

Opening angle measurements are often performed in concert with other mechanical tests, such as tensile testing (Williams et al., 2009; Tian et al., 2011), and in doing so have demonstrated a correlation between OA and arterial stiffness. For example, rabbit carotid arteries exhibit an average OA of 110^∘^, which correlates with a tensile modulus of 2.3 MPa. When the artery is decellularized, the OA decreases to 70^∘^ which correlates with an increase in the tensile modulus to 3.5 MPa (Williams et al., 2009). OA analysis can also be performed after pre-stressing the arteries with a transmural pressure load to cause inflation and deflation and measuring the change in artery diameter and axial force (Matsumoto and Hayashi, 1996; Rachev and Greenwald, 2003). As an example, using OA combined with arterial pre-stress measurements, Matsumoto and Hayashi (1996) reported the mean circumferential stress of the thoracic aorta was 339 kPa for hypertensive rats compared to 263 kPa for normotensive controls, and that the circumferential strains were 0.72 and 0.61, respectively. Although taking an OA measurement is useful when analyzing the residual stress and strain properties of the arterial wall, it limits further testing on the uncut artery.

#### Pulse Wave Velocity Measurements

Pulse wave velocity (PWV) measurements are the primary clinical method used to assess arterial stiffening and to help predict CVD events (Laurent et al., 2006; Van Bortel et al., 2012; Protogerou et al., 2014). A PWV device determines the time for a pressure wave to propagate down a blood vessel by measuring in two separate areas of the body, usually the carotid and femoral arteries. The physical distance between the two catheters is measured on the surface of the body using a tape measure, and the velocity measurement is the ratio of distance traveled to elapsed time (van Popele et al., 2001; Laurent et al., 2006). Measurements along the aorta and aorto-iliac pathway are the most clinically relevant because the aorta and its branches are the first vessels that blood encounters out of the heart and PWV values using this pathway have been shown as predictive values for arterial stiffness (Laurent et al., 2006). The PWV value is directly correlated with the elastic modulus by the Moens–Korteweg equation, which takes into account the vessel radius and the wall thickness and density (Newman and Greenwald, 1978). PWVs are inversely correlated with artery distensibility and compliance (Cecelja and Chowienczyk, 2012). However, PWV does not take into account the differences in arterial stiffness between elastic and muscular parts of the vascular tree (Laurent et al., 2006).

Pulse wave velocity has many advantages over direct mechanical testing because it can be used for clinical applications, is non-invasive, and can directly lead to calculated elastic modulus values. Carotid-femoral PWV measurements are correlated with arterial stiffness and can be used to predict cardiovascular disease in patients (Laurent et al., 1994; Safar et al., 2003). Higher PWV measurements are associated with a higher risk of stroke, cardiovascular death, coronary heart disease (Sutton-Tyrrell et al., 2005) and hypertension (Cecelja and Chowienczyk, 2009). Patients with hypertension and high aortic PWV measurements have increased stenosis frequency, and a higher prevalence of atherosclerotic lesions in the aorta and lower extremities (Maarek et al., 1987). Hallock (1934) used PWV measurements to determine that PWV in the aorta increases from 4.1 m/s in patients ages 5–9 to 6.4 m/s in patients ages 35–44 and up to 10.5 m/s for patients over 65. Notably, PWV increases with age for both men and women (Hallock, 1934; van der-Heijden-Spek et al., 2000), and pulse pressure increases by 25% from ages 30 to 60 (Kelly et al., 1989). Using PWV and distensibility measurements, the 2001 Rotterdam Study demonstrated that stiffness is associated with atherosclerosis in both the carotid artery and the aorta (van Popele et al., 2001). The Rotterdam study also indicated that aortic stiffness alone can be used as a predictor of atherosclerosis because elevated aortic stiffness is associated with atherosclerosis throughout the entire arterial tree (van Popele et al., 2001). PWV has become a powerful method for predicting cardiovascular risk clinically, and in hypertensive patients, it is the best predictor of cardiovascular mortality regardless of age (Blacher et al., 1999).

#### Ultrasound Testing

In addition to PWV measurements, distensibility, and compliance measurements obtained using ultrasound are also used clinically to assess cardiovascular health. Ultrasound non-invasively images internal organs using sound waves and the associated reflected waves. Ultrasound and its accompanying image processing techniques provide a simple and visual determination of arterial mechanical properties. B-mode, or brightness-mode, ultrasound provides a two-dimensional cross-section of the vessel, whereas M-mode, or motion-mode, can record B-mode images in quick succession, providing a ‘video’ of the cross-sectional area. Pulse-Doppler ultrasound records images over time, but also employs the Doppler effect to record blood movement in the vessel. Due to the prevalence of ultrasound equipment, ultrasound is used extensively to produce non-invasive measurements of artery mechanics in both clinical and research settings.

Using ultrasound B- and M-modes, the expansion and contraction of the artery with the cardiac pulse can be measured to determine distensibility, which is a measure of artery elasticity (MacSweeney et al., 1992; Godia et al., 2007). Traditionally, the artery is found using B-mode detection, followed by capturing the movement in the vascular wall over time with M-mode ultrasound. The image of the artery walls is then traced to calculate distensibility from the image series (Godia et al., 2007). The distensibility coefficient is calculated as the relative change in artery diameter divided by the systolic pulse pressure (Reneman et al., 1986). A decrease in artery distensibility is directly correlated with increasing arterial stiffness, and therefore, indicates that distensibility can be used as a metric for cardiovascular disease (Gamble et al., 1994; Selzer et al., 2001).

The clinical definition of compliance is the ratio of blood volume to arterial blood pressure, although in practice, the compliance of an artery can be measured as the ratio of change in cross-sectional area to the change in pressure, which is known as the cross-sectional compliance (Reneman et al., 1986; Tozzi et al., 2000). The cross-sectional compliance value is equal to the distensibility coefficient multiplied by the cross-sectional area of the vessel (Reneman et al., 1986; Gamble et al., 1994). To obtain a compliance measurement, an artery can be examined under pulse-Doppler ultrasound to detect the artery wall movement (Reneman et al., 1986). The expansion and contraction of the vessel are normalized to the zero-load outer diameter and measured in conjunction with an ECG measurement that determines heart rate (Fonck et al., 2007). Techniques in ultrasound software can also assess other cardiovascular risk factors, such as wall strain (Godia et al., 2007), atherosclerotic plaque location, and intima-media thickness (Selzer et al., 2001; van Popele et al., 2001). Introducing microbubbles into the blood stream can allow for contrast enhanced ultrasound, where specific molecular targets in the vessel are imaged to study disease progression (Shim and Lindner, 2014).

Decreased arterial compliance is correlated with a higher risk of cardiovascular disease (Zieman et al., 2005). Using pulse-Doppler ultrasound, Reneman et al. (1986) showed that starting around age 30 the human carotid artery distensibility and cross-sectional compliance decrease. Human common carotid arteries have distensibility coefficients of 30^∗^10^-3^/kPa at age 25 and this decreases linearly to approximately 18^∗^10^-3^/kPa by age 60 (Reneman et al., 1986). The cross-sectional compliance also decreases linearly within this age range from 9^∗^10^-7^ m^2^/kPa to 6.5^∗^10^-7^m^2^/kPa (Reneman et al., 1986). In addition to age-related effects, patients with increased serum cholesterol also have impaired radial artery compliance and distensibility (Giannattasio et al., 1996). A decrease in compliance can be attributed to altered arterial and blood flow pressures due to increases in wall thickening, collagen deposition, or elastin fragmentation (Zieman et al., 2005).

### Microscale Mechanical Tests

**FIGURE 3:**
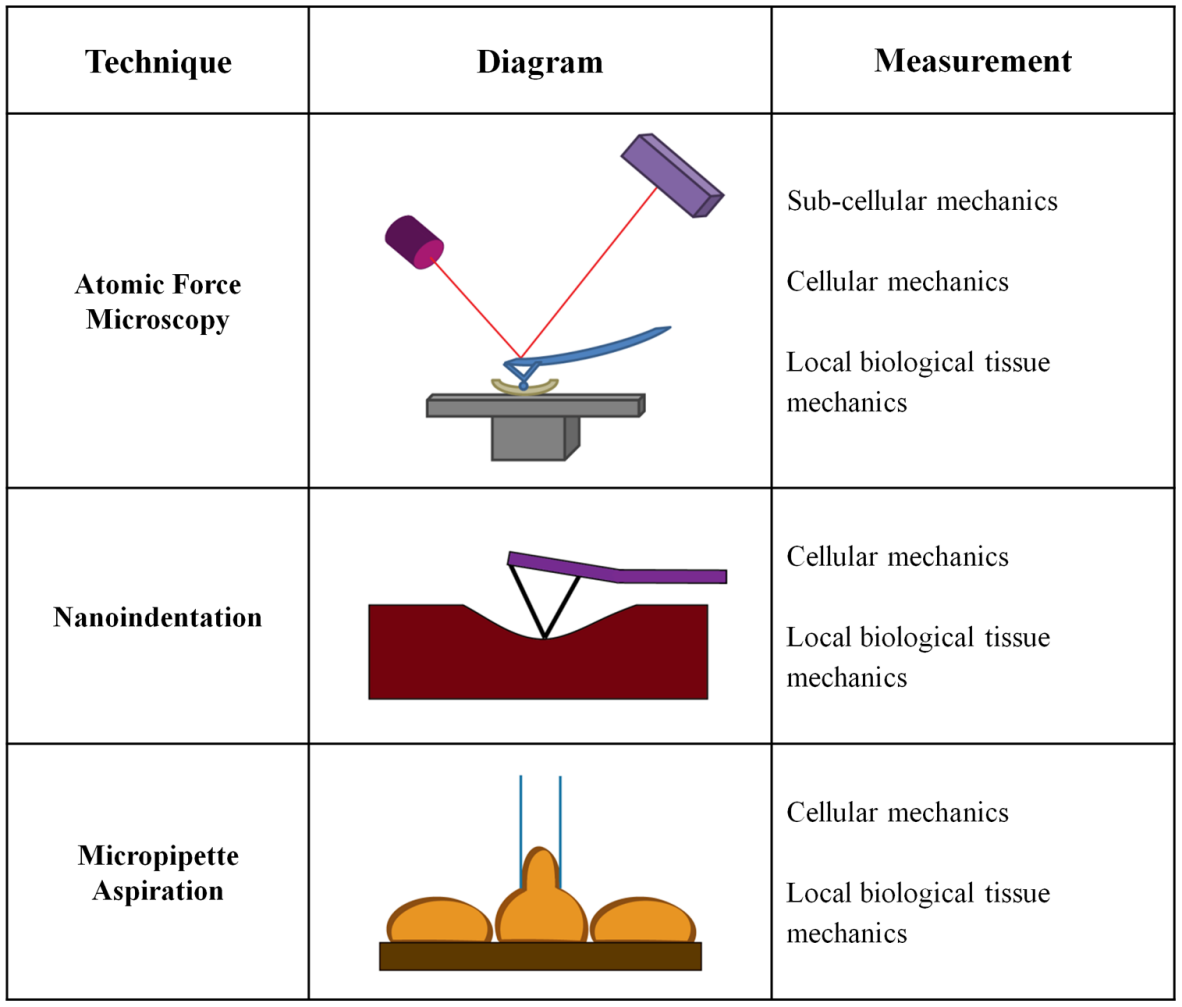
**Microscale techniques to determine artery mechanics**.

While significant emphasis has been placed on macroscale measurements of vessel mechanics, less is known about the changes in the mechanical properties of the individual arterial layers or the ECM within these layers. Macroscale measurements generally treat the artery as a uniform material, and do not take into account that arteries are composite materials with three distinct layers. Mechanical analysis on the cell-scale is fundamental to the field of mechanobiology and our understanding of the mechanical cues driving cell behavior and function. Cell size is on the order of 10s of microns, and therefore macroscale mechanical tests do not necessarily measure the same properties being sensed locally by cells. Several approaches to measure microscale mechanical changes have been developed which measure layer-specific mechanical changes. Microscale tests are more relevant for studies of cellular mechanotransduction in the vessel wall and investigations of the cellular response to changes in arterial mechanics (**Figure 3**).

#### Atomic Force Microscopy

The use of AFM force indentation measurements for ECM mechanical testing in arteries is a relatively newer technique, and it allows for layer-specific mechanical properties to be determined. A cantilever with a micron-scale tip is brought in contact with a sample, and as the cantilever deflects, a laser beam focused on the back of the cantilever is also deflected, providing information regarding the depth of indentation (Binnig et al., 1986; Mann, 2007). The two primary AFM modes are ‘contact mode,’ where the cantilever tip is dragged along the sample surface, or ‘AC (tapping)’ mode, where the tip oscillates near its resonant frequency close to the sample surface and intermittent indentations are recorded (Zhong et al., 1993). Most commonly, AFM is used to “image” samples, creating a topographical map, by dragging the cantilever across a sample (Hurley, 2009). AFM tip materials, geometries, and sizes are dependent on the specific application. AFM indentations for force measurements can use tips with radii from ∼5 to 50 nm, allowing for nanoscale resolution (Hurley, 2009). Spherical beads of varying diameters up to the microscale can also be placed on the AFM cantilever for force indentation measurements (Radmacher, 1997; Costa and Yin, 1999). AFM tips can also be functionalized with biological sensors that limit the interaction area with the material (Leckband, 2011; Chtcheglova and Hinterdorfer, 2013).

There has been an increasing interest in the technique of AFM to obtain force measurements on biological materials (Huynh et al., 2011; Peloquin et al., 2011; Weisbrod et al., 2013). AFM force measurement records the deflection of the cantilever as it indents into the sample material to produce a force versus indentation curve (Binnig et al., 1986). Data can be fit to standard mathematical models such as the Hertz model, to provide mechanical properties such as the elastic modulus at the indentation location (Hertz, 1881; Mahaffy et al., 2000; Peloquin et al., 2011). Within the vasculature, AFM has been used to measure local stiffness at different locations within an artery, as well as the mechanical contributions of specific ECM components, namely collagen and elastin (Beenakker et al., 2012). AFM has also been used to study the mechanics of atherosclerotic plaques and fibrous caps (Hayenga et al., 2011; Marzec et al., 2014), as well the effects of age and diet on intima stiffness (Huynh et al., 2011; Peloquin et al., 2011; Weisbrod et al., 2013).

Importantly, the values found using AFM are orders of magnitude smaller than those found using tensile testing. In porcine aortas, AFM testing showed the tunica adventitia had an elastic modulus range of 0.7–391 kPa and that the pulmonary arteries have a stiffer adventitia that ranges between 2.3 and 1,130 kPa (Grant and Twigg, 2013). Notably, AFM testing of the porcine medial layer indicates it is orders of magnitude stiffer than the adventitia, and has an elastic modulus ranging from 1 to 30 MPa (Beenakker et al., 2012). Furthermore, Lundkvist et al. (1996), reported the intima stiffness of healthy human arteries is 34.4 kPa when measured using AFM, while tensile testing on the composite artery indicates an elastic modulus ranging between 1.5 and 3.8 MPa (Khanafer et al., 2011; Karimi et al., 2013). Bulk mechanics typically provide values of the stiffest layer, the media, and therefore these data underscore the importance of analyzing mechanical properties of individual arterial layers using microscale techniques, especially when studying behaviors of mechanosensitive cells.

Atomic force microscopy has also been used to study cell and sub-cellular mechanics using live samples. AFM can be used to study cellular mechanics, including EC stiffness (Kusche-Vihrog et al., 2011; Lee et al., 2011). It was suggested that cells adopt the relative stiffness values of their matrix; therefore, cell-stiffness may be a measure of vascular health (Byfield et al., 2009). On the subcellular level, AFM has been used to study the properties of the plasma membrane, ECM, and cytoskeleton (Kasas and Dietler, 2008; Lu et al., 2008). When performing sub-cellular analysis, AFM tips can be modified to create bio-specific molecular sensors that are used for studying surface receptor interactions and measuring adhesion molecule stiffness (Leckband, 2011; Sarangapani et al., 2011). Simultaneous topography and recognition imaging (TREC) is a recent advancement in AFM technology, where a ligand is functionalized to the AFM cantilever tip to provide simultaneous measurements of material topography and cell-ligand interactions (Preiner et al., 2009). A recent study using TREC with a VE-cadherin-Fc modified AFM tip showed VE-cadherin forms ellipsoid clusters and suggests the protein is bound to actin filaments (Chtcheglova and Hinterdorfer, 2013). Because AFM can be used to determine artery mechanics at the cellular and subcellular levels, and its applications continue to develop, it is expected to have increased prevalence in the mechanobiology field.

#### Nanoindentation

Nanoindentation measurements use a nano-scale tip to indent into a material and generate a load versus displacement curve that can be used to calculate material mechanics, such as stiffness. Unlike AFM which records cantilever displacements, in nanoindentation, an external load is applied to the indenter tip to push the tip into the surface (Mann, 2007). Displacement in nanoindentation is measured directly by the cantilever’s depression into the sample surface, unlike the deflected laser measurement used by the AFM. Whereas older studies relied on imaging the tip depression in the sample to determine the area of contact, current nanoindentation methods can estimate the area of contact based on a well-defined tip geometry, such as a triangular Berkovich or spheroid tip (Oliver and Pharr, 2004). Nanoindentation allows for micro- and nanoscale measurements, with a force range of 1 μN to 500 mN, and a displacement range of 1 nm to 20 μm (Fischer-Cripps, 2002; Ebenstein and Pruitt, 2006). Nanoindentors have indentation depths on the nanometer scale (Fischer-Cripps, 2002); however, their contact area with the material can be hundreds to thousands of nanometers wide (Hurley, 2009). These length scales bridge the gap between more sensitive AFM measurements and macroscale testing.

Material hardness is calculated using nanoindentation by dividing the load on the tip during indentation by the contact area, and the plastic unloading stiffness is the slope of the load versus displacement curve at a given point (Oliver and Pharr, 2004). Different mathematical models are used to fit nanoindentation data and include the Hertz model (Hertz, 1881; Johnson, 1982), the Oliver-Pharr model (Oliver and Pharr, 1992, 2004), and the Johnson–Kendall–Roberts (JKR) adhesion model (Johnson et al., 1971). The choice of analysis model is based on the properties of the tip-sample interaction, taking into account the shape of the indentation curve and any discontinuities in the curve (Mann, 2007). For biological tissues and hydrated biomaterials, significant adhesion between the tip and sample must be accounted for when determining mechanical properties with nanoindentaiton (Kohn and Ebenstein, 2013).

Many biological materials have been characterized using nanoindentation, including bone and cartilage as well as biomimetic materials such as mollusk shells and spider silk (Ebenstein and Pruitt, 2006). Arterial tissue probed with nanoindentation indicates that porcine elastic aortas have elastic moduli between 60 and 70 kPa (Hemmasizadeh et al., 2012). The technique has also been used to measure the mechanical properties of rat arteries, where the effective elastic moduli ranged from 22 to 37 kPa (Hanna et al., 2013). The stiffness of carotid plaques has also been measured using nanoindentation (Ebenstein et al., 2009). The arterial stiffness values obtained with nanoindentation are comparable to AFM measurements, and both are orders of magnitude lower than the bulk vessel stiffness.

Although nanoindentation and AFM can provide similar information, nanoindentation is a more direct measurement technique for local mechanical property determination. Both methods produce force versus indentation curves to calculate mechanical properties such as the elastic modulus, however, the AFM has increased complexity that relies not only on the indentation parameters, but also on a laser deflection measurement. However, AFM is advantageous for smaller scale biological applications and has greater versatility in the information it can provide.

#### Micropipette Aspiration

Micropipette aspiration is a less complex technique to measure the mechanics of cells and tissues when compared to AFM and nanoindentation. During testing, a pipette uses suction to pull one end of a cell or matrix into a 0.5–0.8 mm glass tube (Sato et al., 2000; Matsumoto et al., 2002). The distance the material travels into the pipette is tracked using light microscopy (Shao et al., 1998) and is directly related to the material stiffness. Suction forces as low as 1 nN were used to measure the elastic modulus of ECs, which were reported to be 0.5 kPa (Hochmuth, 2000) and increased with increasing substrate stiffness (Byfield et al., 2009).

Soft biological tissue mechanics are measured using pipette aspiration by applying an aspiration pressure on the inside of the pipette to deform the tissue (Aoki et al., 1997). Based on the pipette properties and the degree of deformity, the tissue elastic modulus is calculated. Micropipette aspiration has been used to measure the ECM properties of porcine and rabbit aortas (Sato et al., 2000; Matsumoto et al., 2002; Ohashi et al., 2005). The rabbit aortic arch has an elastic modulus between 40 and 50 kPa compared to an elastic modulus of ∼40 kPa for the thoracic aorta (Matsumoto et al., 2002). The elastic moduli measured using micropipette aspiration are similar to the values obtained using AFM and nanoindentation. Although micropipette aspiration is relatively simple to perform and is well-characterized, it is a lesser used technique for testing the mechanics of *ex vivo* samples. Newer mechanical testing techniques such as nanoindentation and AFM have become more popular likely because of their versatility in measuring cellular and sub-cellular properties.

## Effects of Vascular Mechanics on Atherosclerosis

### Arterial Stiffening Promotes Vascular Diseases

The mechanical properties of arteries are altered with both age and disease primarily due to changes in the ECM. Vessel stiffness measurements using PWV and ultrasound are widely used in the clinic as indicators of vascular health, but the importance of vascular stiffening, especially on the microscale, in promoting disease is not well-understood (O’Rourke et al., 2002). Recent data suggest numerous cell types respond to matrix stiffness and that stiffness can promote stem cell differentiation, tissue morphogenesis, and tumor malignancy (Paszek and Weaver, 2004; Sieminski et al., 2004; Paszek et al., 2005; Engler et al., 2006). As such, it is likely that the stiffening, which occurs in the vasculature with age and disease, promotes cell dysfunction.

During atherosclerosis, cholesterol permeates into the blood vessel wall and must be retained there to elicit the inflammatory reaction resulting in plaque formation (Libby, 2002). Age-related changes in the arteries caused by ECM rearrangements directly contribute to increased LDL accumulation but they also alter endothelial and VSMC function to contribute to atherogenesis (Tada and Tarbell, 2004; Rudijanto, 2007; Kwon et al., 2008; Huynh et al., 2011). Stiffer arteries have been shown to exhibit increased permeability *in vivo*, and therefore are thought to have greater cholesterol uptake and atherosclerotic plaque initiation (Huynh et al., 2011).

Animal studies by Kothapalli et al. (2012) recently demonstrated that arterial stiffening and ECM crosslinking precede cardiovascular disease development. Treatment with the lysyl oxidase inhibitor BAPN to prevent collagen crosslinking reduced arterial stiffness and decreased atherosclerotic plaque size ∼50% in ApoE mice. A decrease in arterial stiffness reduced the deposition of a pro-atherogenic fibronectin and collagen ECM by VSMCs and limited monocyte binding. It is also known that media calcification precedes atherosclerotic plaque development, and there is a strong relationship between arteriosclerosis and the calcification and degeneration of elastic fibers in arteries (Blumenthal et al., 1944; Shekhonin et al., 1985). Both human clinical trials using PWV macroscale measurements and animal studies using AFM microscale measurements of the arterial intima have demonstrated that hypertension is preceded by vessel stiffening (Kothapalli et al., 2012; Quevedo, 2012). Furthermore, in hypertensive patients, increased arterial stiffness is associated with increased mortality rates (Laurent et al., 2001). Mounting evidence suggests that arterial stiffness, measured on both macro- and microscales are causes of vascular pathologies, rather than consequences as previously hypothesized.

### Mechanical Cues Affect Endothelial Cells to Promote Atherogenesis

Endothelial cells are known to be mechanosensitive, and are important regulators of vascular health and atherogenesis. The effect of flow on EC behavior has been widely studied, and it is well-established that ECs align in the direction of flow, that laminar flow promotes an atheroprotective endothelium, and that atherosclerotic plaques develop predominantly in regions of disturbed flow (Caro et al., 1969; Zarins et al., 1983; Levesque and Nerem, 1985; Tzima et al., 2005). The ECM composition has also been shown to alter EC behavior including increased RhoA activity, ICAM-1 expression, proliferation, morphology, and adhesion (Ingber et al., 1990; Tzima et al., 2001, 2002; Young et al., 2007) Recently, it has become apparent that ECs also respond to ECM mechanical cues where increased matrix stiffness has deleterious effects on endothelial NO production and barrier integrity (Huynh et al., 2011; Kohn et al., 2015).

An important function of the vascular endothelium in atherosclerosis prevention is acting as a protective barrier against cholesterol accumulation and leukocyte transmigration into the intima (Schwenke and Carew, 1989). Using AFM, our group has shown the arterial intima stiffens with age, and that in response to increased ECM matrix stiffness, EC-cell junction size and permeability increase both *in vitro* and *in vivo* (Huynh et al., 2011). Mechanistically, increased substrate stiffness activates the Rho/Rho kinase pathway to increase cellular contractile forces and compromise cell-cell junction integrity (Huynh et al., 2011; Krishnan et al., 2011). Recent work by Szulcek et al. (2013) demonstrated the complexity of endothelial monolayer regulation. RhoA is involved in both monolayer gap formation and closure depending on its subcellular localization. Immune cell infiltration into the intima has important implications for driving atherosclerotic plaque formation, and the increased EC-cell junction size in response to substrate stiffness facilitates leukocyte transmigration by the predominant paracellular route (Huynh et al., 2011; Stroka et al., 2013). Notably, the endothelial expression of VCAM-1, ICAM-1, and E-selectin were unaffected by substrate stiffness *in vitro*, suggesting the changes were largely driven by mechanics. In addition to the endothelial response to matrix mechanics compromising the arterial barrier against leukocyte transmigration, recent studies show that leukocytes also respond to substrate mechanics. Leukocytes exhibit elevated transmigration behavior with increasing stiffness and biphasic migration speeds (Hayenga and Aranda-Espinoza, 2013; Stroka et al., 2013). Interestingly, AGEs can promote increased adhesion molecule expression in ECs, suggesting that arterial stiffening caused by collagen glycation may have the synergistic effects of increased substrate mechanics and increased adhesion molecule expression contributing to leukocyte transmigration.

### Arterial Stiffening Alters VSMC Behavior to Promote Atherosclerosis

A phenotypic switch from a contractile to a motile, synthetic phenotype in VSMCs is characteristic of VSMCs found in atherosclerotic lesions. During atherogenesis, VSMCs migrate from the media into the intima where their proliferation and ECM secretions increase plaque size (Rudijanto, 2007). Interestingly, VSMCs are durotactic, preferentially migrating in the direction of increased substrate stiffness, but their maximum migration speed depends on both matrix stiffness and ECM chemical cues (Peyton and Putnam, 2005; Isenberg et al., 2009; Sazonova et al., 2014). A proliferative phenotype was observed by VSMCs cultured on stiff collagen fibrils when compared to compliant collagen gels, even though β_1_ integrin mediated cell-ECM interactions were comparable on both substrates (McDaniel et al., 2007). Similar to migration, VSMC proliferation is also dependent on ECM ligand density, but it has been demonstrated that increased matrix stiffness is the dominant factor affecting proliferation (Vatankhah et al., 2014). In addition to ECM cues, VSMC proliferation and migration is also stimulated by platelet derived growth factor (PDGF) released by ECs and macrophages during atherogenesis. Interestingly, increased matrix stiffness enhances VSMC proliferation and migration induced by PDGF signaling (Brown et al., 2010; Huynh et al., 2013).

### Interventions for Age-Related Cardiovascular Stiffening

Stiffening of the arteries with age is well-established, and it is now becoming apparent that arterial stiffness contributes to cardiovascular diseases; therefore, preventing or reversing stiffening may be one method to improve cardiovascular health. Lifestyle changes and pharmacological interventions are two strategies for decreasing arterial stiffness (**Table 1**). Exercise is the most well-studied lifestyle intervention to improve arterial compliance.

**Table 1:** **Selected interventions with human studies to reduce arterial stiffness**.

Category	Intervention	Study	Outcome	Reference
Lifestyle	Aerobic exercise	Brisk walking in healthy sedentary male adults	Increased central arterial compliance	Tanaka et al. (2000)
Pharmaceutical AGE blocker	Aminoguanidine	Phase 3 clinical trial	Early termination due to safety and efficacy	Freedman et al. (1999) and Thornalley (2003)
Pharmaceutical AGE breaker	Alagebrium chloride (ALT-711)	Phases 2 and 3 clinical trials	No increase in cardiovascular health (Lifetime Risk Score) Clinical trials terminated due to finances	Oudegeest-Sander et al. (2013); clinicaltrials.gov
Pharmaceutical AGE breaker	TRC4186	Phase 1 clinical trial	Safety, tolerance, and pharmacokinetics established	Chandra et al. (2009)

#### Aerobic Exercise Reduces Macroscale Arterial Stiffening

Numerous human studies have demonstrated that habitually active adults have more compliant arteries when compared to their age-matched sedentary peers and that moderate aerobic exercise interventions successfully increased macroscale arterial compliance in healthy aged populations (Tanaka et al., 2000; Seals et al., 2008; Santos-Parker et al., 2014). Brisk walking can improve arterial compliance in as little as 3 months in healthy middle aged men (Tanaka et al., 2000). However, in aged individuals with hypertension the same gains in arterial compliance were not achieved by a short-term aerobic exercise intervention, suggesting that existing cardiovascular pathologies may limit the benefits of exercise on arterial stiffness (Ferrier et al., 2001). The mechanism by which exercise restores arterial compliance is not fully understood and appears to vary with the type of exercise. Mouse studies show that voluntary wheel running decreases Type I collagen levels in the media and adventitia of old mice, while studies involving swimming and treadmill running did not show changes in arterial collagen composition (Nosaka et al., 2003; Maeda et al., 2005; Fleenor et al., 2010). Exercise may increase collagen turnover, thus preventing the accumulation of AGEs. However, while increased markers of collagen synthesis and turnover have been found in response to exercise for cardiac, tendon, and bone collagens, studies with arterial collagen still need to be completed (Langberg et al., 1999; Wallace et al., 2000; Radauceanu et al., 2007). It is important to note that deposition of collagen in bone and tendon in response to exercise is indicative of beneficial strengthening while collagen deposition in the arteries contributes to pathological stiffening, therefore, it is possible that the effects of exercise on collagen composition may be tissue specific.

#### Pharmacological Prevention of AGE Accumulation

Pharmacological interventions for arterial stiffening primarily fall into two categories: AGE blockers and AGE breakers. Several chemical compounds to prevent arterial stiffening by inhibiting AGE accumulation have been studied, but thus far, none have made it past clinical trials. Aminoguanidine was the first well-studied inhibitor of AGEs. Early studies in diabetic rats showed that it was able to inhibit AGE formation by blocking carbonyl reactivity on early glycation products even when animals were fed high glucose diets (Brownlee et al., 1986). Later studies with an aged, non-diabetic rat model showed aminoguanidine prevented arterial stiffening in 24-month old, end-of-life rats without altering collagen content (Corman et al., 1998). Despite the early promise of aminoguanidine, clinical trials were terminated when it caused impaired liver functionality and the initiation of lupus-like illnesses in patients (Freedman et al., 1999). An alternative AGE inhibitor, 2,3 diaminophenazine (2,3 DAP) did not make it past pre-clinical toxicity tests (Soulis et al., 1999). ALT-946 and OPB-9195 are other examples of AGE blockers that possess similar hydrazine structures to aminoguanidine, that have not yet reached clinical trials. Initial data indicates that ALT-946 is more effective and less toxic than aminoguanidine (Forbes et al., 2001; Wada et al., 2001). The failure of the AGE inhibitors mentioned here, and others, to succeed in clinical trials more than 15 years after initial studies with aminoguanidine, highlights the difficulty of safely and effectively reducing AGE accumulation *in vivo*.

#### Pharmacological Breaking of AGE Crosslinks

The second class of pharmaceutical interventions used to overcome arterial stiffness are compounds that break AGE crosslinks after they have been formed. The most widely studied of these is the thiazelium based ALT-711 (alagebrium chloride), which was discovered as a more stable form of the very first AGE breaker, PTB (Vasan et al., 1996). These nucleophilic compounds break carbon–carbon bonds between adjacent carbonyl groups in crosslinked proteins (Vasan et al., 1996; Kim and Spiegel, 2013). ALT-711 showed efficacy *in vitro* and *in vivo* at cleaving AGE crosslinks. The resulting collagen fragmentation increased the elasticity of cardiac and arterial tissue (Vasan et al., 1996; Wolffenbuttel et al., 1998; Little et al., 2005). The early success of ALT-711 led to more than 12 combined Phases 2 and 3 clinical trials ^2^. Despite the potential for ALT-711, a randomized factorial study comparing the interaction between ALT-711 and exercise in older (over age 70) sedentary adults showed no improvement in arterial stiffness measured with PWV. Cardiovascular health measured using the Lifetime Risk Score (LRS) improved with exercise, but not the drug intervention (Oudegeest-Sander et al., 2013). All ongoing clinical trials for ALT-711 were terminated in 2009 by Synvista Therapeutics Inc. for financial reasons.^2^ The safety, pharmacokinetics, and tolerance of another AGE crosslink breaking compound, TRC4186, were established during a successful phase 1 clinical trial and published in 2009 (Chandra et al., 2009). However, the necessary phase 2 and 3 clinical trials showing efficacy and continued safety have not been completed. Studies on a new, safer AGE breaker, C36 (3-benzyloxycarbonylmethyl-4-methyl-thiazol-3-ium bromide) showed decreased systemic arterial stiffness and improved collagen composition in diabetic rats (Cheng et al., 2007). There currently are no AGE blockers or breakers on the market today, indicating the difficulty of successfully and safely overcoming the effects of tissue stiffening that occur with age.

## Future Perspectives

As the contribution of arterial stiffening to cardiovascular diseases comes into better focus, new studies will need to rely on macroscale measurement techniques, and expand the microscale understanding of how stiffening of each arterial layer contributes to disease. Recently, Weisbrod et al. (2013) have shown that intimal stiffening precedes hypertension and Huynh et al. (2011) showed that intimal stiffening leads to compromised endothelium integrity. These new findings complement existing literature with macroscale measurements that emphasize stiffening of the adventitial and medial layers. A particular challenge will be to develop microscale tools that can measure intimal stiffening *in vivo* for use in human clinical diagnoses and to contribute to a better understanding of the underlying mechanisms that govern the relationship between arterial stiffening and cardiovascular disease.

## Conflict of Interest Statement

The authors declare that the research was conducted in the absence of any commercial or financial relationships that could be construed as a potential conflict of interest.

## Author Contributions

JK and ML collected and analyzed relevant literature, wrote the manuscript, and created the figures and table. CR designed the research topic and wrote the manuscript.

## Footnotes

1 http://www.who.int/nmh/publications/ncd_report_full_en.pdf

2 http://clinicaltrials.gov/
